# A hydrocele revealing epididymal tuberculosis

**DOI:** 10.1099/acmi.0.000781.v4

**Published:** 2025-09-10

**Authors:** Yassine Ben Lahlou, Zakaria Laanibi, Zakaria Malihy, Elmostapha Benaissa, Adil Maleb, Abderrahim Elktaibi, Mariama Chadli, Mostafa Elouennass

**Affiliations:** 1 Bacteriology Department, Mohammed V Military Training Hospital, Rabat, Morocco; 2 Bacteriology Department, Mohammed VI University Hospital, Oujda, Morocco; 3 Pathology Department, Mohammed V Military Training Hospital, Rabat, Morocco

**Keywords:** epididymal tuberculosis, hydrocele, urogenital tuberculosis

## Abstract

Genitourinary tuberculosis is a severe form of extrapulmonary tuberculosis. The kidneys are the most commonly affected organs, followed by the epididymis, testicles, bladder, ureter and prostate. Notably, epididymal tuberculosis is one of the forms of genital tuberculosis presenting with specific clinical features, which may include epididymitis, orchid-epididymitis or hydrocele. We report the case of a patient with a hydrocele that revealed epididymal tuberculosis. Utilizing molecular biology techniques, a diagnostic test for epididymal tuberculosis was established. The patient was treated conservatively with tuberculosis medication for 6 months.

## Data Summary

No data were reused or generated.

## Introduction

Tuberculosis is a public health problem, particularly in developing countries. Genitourinary tuberculosis, known as a severe variant of tuberculosis, accounts for 20–73% of all extrapulmonary cases [1]. Among the affected organs, the kidneys are most commonly affected. However, epididymal localization remains relatively rare [2].

We report the case of a patient with a hydrocele that revealed epididymal tuberculosis.

## Case presentation

### Patient’s information and timeline

A 70-year-old male patient, an active smoker, was referred by his general practitioner because of a clinical finding of mild pain in the left scrotum that had been persistent for a month. His medical history was notable for chronic obstructive pulmonary disease and rheumatoid arthritis on methotrexate 2.5 mg once per week.

### Clinical findings

Our patient was in good general condition. There was no fever, night sweats or other constitutional symptoms of tuberculosis. Physical examination revealed no signs of hernia and a soft hypogastrium. The appearance of the penis was normal, while the scrotum had fluid accumulation. Scrotal ultrasonography showed a significant amount of fluid around the left testicle and a smaller amount around the right testicle. Surgical treatment was then decided, and a unilateral epididymectomy was performed intraoperatively.

### Laboratory findings

Cytobacteriological examination of urine was sterile but showed significant leukocyturia and haematuria of 37.10^3^ ml and 13.10^3^ ml, respectively. Complete blood count revealed haemoglobin of 14 g dL^−1^, platelets of 183,000 G l^−1^ and white blood cells of 7,475/mm^3^ (neutrophils 60%, lymphocytes 37%).

A molecular biology test (GenXpert®) on the epididymectomy sample enabled the detection of *Mycobacterium tuberculosis* without detecting resistance to rifampicin.

**Fig. 1.**
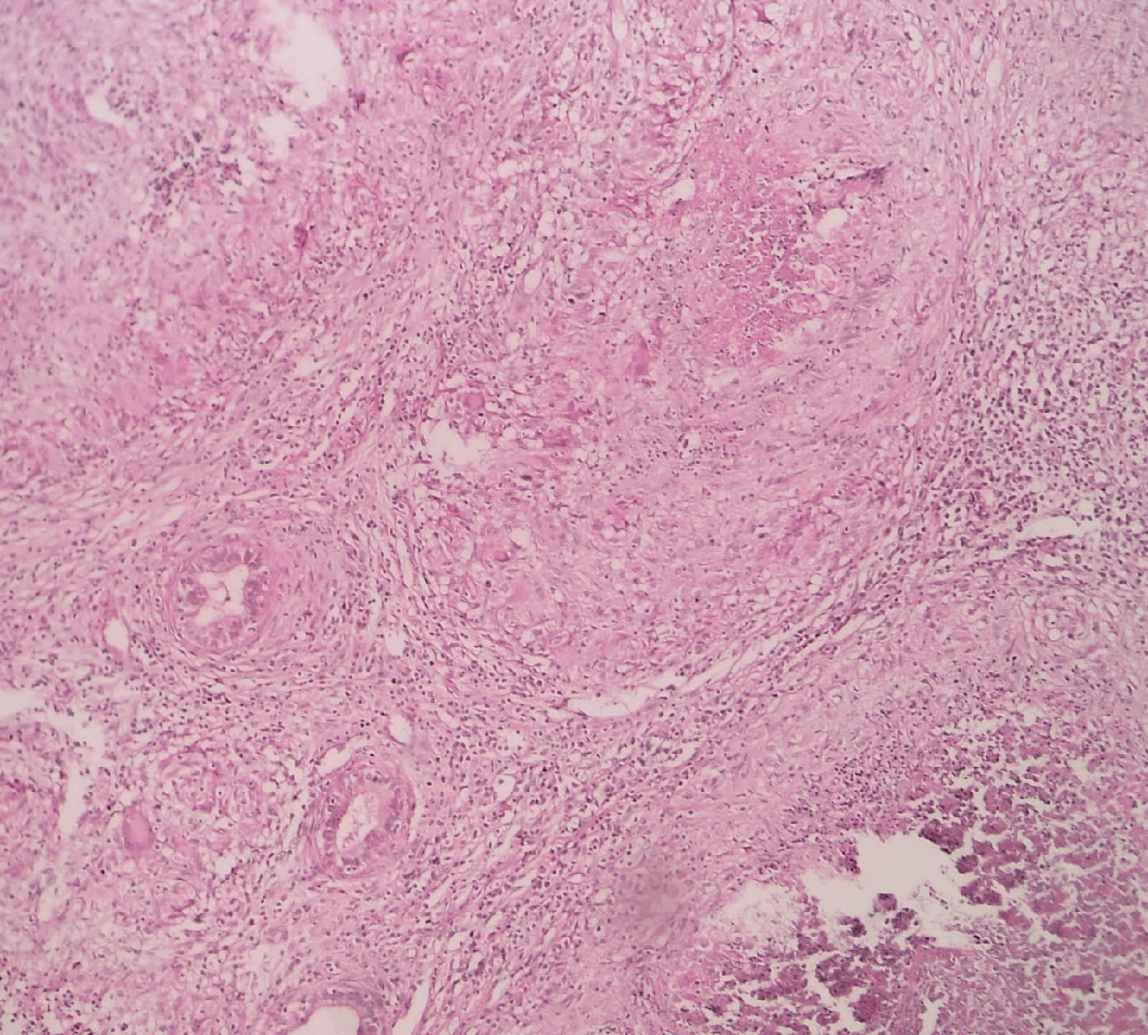
Epididymal parenchyma with a tuberculoid necrotizing granuloma adjacent to tubules. Haematoxylin eosin ×200.

The direct examination using Ziehl–Neelsen stain was negative, and the culture on solid Löwenstein–Jensen media also yielded a negative result after 2 months of incubation. The test for *M. tuberculosis* in the urine was requested but not performed. An anatomopathological examination of the epididymectomy specimen concluded that it was a caseous-follicular granulomatous epididymitis consistent with a tuberculous origin (Fig. 1).

### Treatment and follow-up

Antituberculous treatment with rifampicin (450 mg), isoniazid (300 mg), ethambutol (800 mg) and pyrazinamide (1500 mg) was started for 2 months before a 4-month continuation period of treatment with rifampicin (450 mg) and isoniazid (300 mg). The patient’s clinical course showed gradual improvement.

## Discussion

Tuberculosis remains a significant global public health problem. The World Health Organization reports that more than 10 million people develop active tuberculosis each year and 1.6 million deaths are attributed to the disease [3]. Genitourinary tuberculosis is considered a severe form of tuberculosis and accounts for 20–73% of all extrapulmonary cases [1,4, 5]. The kidneys are the most frequently affected organ, followed by the epididymis, testicles, bladder, ureter and prostate. In Morocco, nearly 30,000 cases of tuberculosis, including all forms, are recorded annually. Genitourinary tuberculosis ranks as the fifth most common form of tuberculosis in the country, following lung, lymph node, digestive and osteoarticular involvement [6].

The manifestations of genital tuberculosis in males can vary, with epididymitis [7,9] or orchid epididymitis [10] being common. However, it can also manifest itself in the presence of a common hydrocele [7,9, 11, 12] or as a pseudotumoral appearance. In our patient, hydrocele was the predominant symptom that led to consultation. Considering that we are in a tuberculosis-endemic country and observing a sterile cytobacteriological urine examination with significant leukocyturia and haematuria (37.10^3^ ml^−1^ and 13.10^3^ ml^−1^, respectively), the primary diagnosis to exclude was tuberculosis. Therefore, this diagnosis can be challenging [13] due to the variability of clinical symptoms. Biologically, haematuria and/or leukocyturia are commonly observed without isolation of specific bacteria on standard culture media, similar to our patient.

In most cases, the involvement is one-sided [2,7]. This was the case for our patient. While the average age of onset is typically between 38 and 40 years [2], tuberculosis can affect people of all ages, including children. Risk factors such as immunosuppression, smoking and alcoholism increase susceptibility to genitourinary tuberculosis. Our patient has two risk factors related to smoking and immunosuppressive therapy.

Several theories have been put forward about the route of infection of the epididymis in tuberculosis. While the ductal route, in which infection ascends along the sperm route from the prostate and seminal vesicles, plays a role, haematogenous spread may also be responsible for cases of tuberculous epididymitis without renal involvement or *M. tuberculosis* detection in urine [14]. In our case, we could not prove the connection with urinary tuberculosis because the patient did not bring urine for testing. Lymphatic involvement is also recognized [2]. In rare cases, tuberculous epididymitis can be the result of sexual transmission.

In our case, molecular biological tests provided a definitive diagnosis. This technique is extremely valuable due to its high sensitivity, specificity and rapid results compared to conventional methods, especially for paucibacillary samples, facilitating rapid treatment and preventing complications [15]. While the most common complication of epididymal tuberculosis is the possible impairment of fertility due to seminiferous tract obstruction or testicular necrosis due to caseous necrosis [7,13], it can also lead to serious, life-threatening complications such as psoas abscess and Addison’s disease [16].

Regarding treatment, in addition to unilateral epididectomy, our patient received medical treatment according to the Moroccan national tuberculosis protocol with rifampicin, isoniazid, pyrazinamide and ethambutol [17]. Some authors report success with treatment with rifampicin injection into the testicular vagina, which allowed higher concentrations to be achieved in contact with the lesion [13].

## Conclusion

The case presented highlights the importance of considering tuberculous epididymitis as a possible diagnosis when hydrocele occurs, particularly in an endemic setting. It emphasizes the value of using molecular biology tests in such cases to enable accurate detection of *M. tuberculosis*.
